# 

**Published:** 1900-07-14

**Authors:** 


					rrijy nxjanranr. - ? ?? A ne sianuai ir ui mgiicsi puiny* ?juuncei. uui^x i*in, hw,
CADBURY'S r+> U lji CADBURY'S
rnnnfl im. M\ JMc* - a
cocoa cocoa
ABSOLUTELY PURB. ^ NO DRUGS OR ALKALIES USED.
No. 720. Vol. XXVIII. Satobdat,
July 14, 1900.
-SubBcriptonTenns.J pRICE TWOPENCE,

				

